# On the Importance and Dangers of Rest in Pulmonary Consumption

**Published:** 1874-02

**Authors:** 


### On the Importance and Dangers of Rest in Pulmonary Consumption.

An interesting paper on this subject, by Dr. Berkart has drawn from Dr. Horace Dobell a communication on this topic, which appears in *The British Med. Jour.* of Nov. 22. He says: "The rules for the cautious application of localized rest in lung-diseases which I recommended, as dictated by a consideration of the nature of tuberculosis, and justified by the results of my own practice, are as follows:

"1. If one lung, or a portion of one lung, or a portion of each lung, has become diseased, under circumstances which makes it certain that there is no constitutional cause of lung-disease, then it is safe to secure localized rest for the diseased part, and to throw the extra work upon the sound parts; but even then it is necessary to be cautious that the extent of the lung so rested is not too large in proportion to the extent of sound lung upon which the extra work is thrown. If there is any question about this, rest of the whole body must be secured in addition to the localized rest of lung, so as to save the sound lung from as much work as possible.

"2 If there is a constitutional cause of lung-disease, but only a small area of lung at present suffering, and that in the upper lobes, while there is a capacious chest with large areas of lung in the lower portions quite sound and insufficiently used, then it is safe to secure localized rest for both upper lobes, and to make the lower portions do a fairer proportion of the work; but even under these circumstances, the respirations should be kept at as low a point as practicable.

"3. If a portion of lung has become disintegrated, under the influence of constitutional causes, and remains obstinately unhealed after all constitutional symptoms have been arrested, and, for some time past, no other portions of lung have shown a tendency to yield, then I think it is quite safe to secure localized rest for the disintegrated portion, so as to give it a fairer chance for healing; while an amount of air and exercise may be allowed to the patient, for the purpose of improving his reparative powers, which could not have been permitted while the damaged lung was exposed to the same amount of action as the sound parts. But even here the utmost caution is required not to carry the exercise beyond a very limited amount.

"4. If the constitutional tendency to lung-disease—the abnormal physiological state—is strong, and signs of impending mischief in the lungs are scattered, no localized rest should be attempted, but every means should be brought to bear upon the important object of maintaing respiration at its lowest point consistent with life and nutrition, until the constitutional tendency has become passive and the local symptoms have been removed.

"In conclusion, to prevent misapprehension on so vital a point, let me remind my readers that, in urging 'the importance of rest in consumption,' I am referring to cases where the lungs are already damaged, or in which the constitutional disease has declared itself in sufficient force to render tuberculization imminent. If the symptoms are only what are commonly called premonitory, that is, if they are those of commencing tuberculosis, and no reason or sign is discoverable which justifies the suspicion that tuberculization has commenced; if a sufficiency of fat remains without calling upon the albumenoid tissues, the principles of treatment are quite opposite to those detailed."—*Medical Record.*

